# EGR1 regulates cellular metabolism and survival in endocrine resistant breast cancer

**DOI:** 10.18632/oncotarget.18292

**Published:** 2017-05-30

**Authors:** Ayesha N. Shajahan-Haq, Simina M. Boca, Lu Jin, Krithika Bhuvaneshwar, Yuriy Gusev, Amrita K. Cheema, Diane D. Demas, Kristopher S. Raghavan, Ryan Michalek, Subha Madhavan, Robert Clarke

**Affiliations:** ^1^ Department of Oncology, Lombardi Comprehensive Cancer Center, Georgetown University Medical Center, Washington, DC, USA; ^2^ Innovation Center for Biomedical Informatics (ICBI), Georgetown University Medical Center, Washington, DC, USA; ^3^ Department of Biostatistics, Bioinformatics and Biomathematics, Georgetown University, Washington, DC, USA; ^4^ Metabolon, Inc, Durham, NC, USA

**Keywords:** breast cancer, endocrine resistance, transcriptomics, metabolomics

## Abstract

About 70% of all breast cancers are estrogen receptor alpha positive (ER+; ESR1). Many are treated with antiestrogens. Unfortunately, *de novo* and acquired resistance to antiestrogens is common but the underlying mechanisms remain unclear. Since growth of cancer cells is dependent on adequate energy and metabolites, the metabolomic profile of endocrine resistant breast cancers likely contains features that are deterministic of cell fate. Thus, we integrated data from metabolomic and transcriptomic analyses of ER+ MCF7-derived breast cancer cells that are antiestrogen sensitive (LCC1) or resistant (LCC9) that resulted in a gene-metabolite network associated with EGR1 (early growth response 1). In human ER+ breast tumors treated with endocrine therapy, higher EGR1 expression was associated with a more favorable prognosis. Mechanistic studies showed that knockdown of EGR1 inhibited cell growth in both cells and EGR1 overexpression did not affect antiestrogen sensitivity. Comparing metabolite profiles in LCC9 cells following perturbation of EGR1 showed interruption of lipid metabolism. Tolfenamic acid, an anti-inflammatory drug, decreased EGR1 protein levels and synergized with antiestrogens in inhibiting cell proliferation in LCC9 cells. Collectively, these findings indicate that EGR1 is an important regulator of breast cancer cell metabolism and is a promising target to prevent or reverse endocrine resistance.

## INTRODUCTION

Resistance to endocrine therapy is a major clinical problem for the management of estrogen receptor positive (ER+) breast cancers. ER+ tumors comprise 70% of all breast cancer cases. Antiestrogens such as tamoxifen, ICI 182,780/faslodex/fulvestrant (ICI) or aromatase inhibitors (AI) are widely used endocrine therapies but little is known about the complex cellular pathways that contribute to resistance [1, 2]. Deregulation of metabolic pathways regulated by oncogenes such as MYC has been implicated in endocrine resistant breast cancer [3–5]. However, to understand the systems-level changes in endocrine resistance, biologically relevant interactions between genes and metabolites need to be identified and validated. Using paired cell lines that are either sensitive or resistant to antiestrogens we generated and integrated data from transcriptomics (microarray analysis) and metabolomics (GC/MS and UPLC/MS). Within our gene-metabolite integrated model, we selected to further study the role of EGR1 (early growth response 1), a gene that is known to be deregulated in some cancers [6].

EGR1 is an immediate-early gene induced by estrogen, growth factors, or stress signals, and can exhibit both tumor suppressor and promoter activities. A nuclear phospho-protein and transcription factor that can promote cell proliferation and cell death [7, 8], EGR1 can be induced by external stimuli, with its induction being either transient or sustained. Such a diverse array of functions is achieved through differential regulation of EGR1 expression and its selection of target genes [7]. A highly conserved DNA-binding domain on EGR1 targets the GC-rich consensus sequence GCG (G/T) GGGCG. Transcriptional activity of EGR1 is further regulated by NAB-1 and NAB-2 (NGF-I A-binding proteins) [9, 10]. EGR1 can promote growth of some hormone regulated cancers including prostate cancer [11]. EGR1 mediated signaling is important for the normal development of female reproductive organs [12] but its precise role in breast cancer remains unclear. The EGR1 gene is deleted in some ER-negative breast tumors [13]. In ER+ breast cancer cells, EGR1 is induced by estrogen treatment following raf-1 kinase activation [14] and is inhibited with acquired resistance to ICI [15]. EGR1 has been associated with sensing cellular glucose levels [16, 17], in fatty acid metabolism and inflammation [18] in various cells. In this study, we investigated the role of EGR1 in endocrine resistance to validate an integrated model consisting of differentially expressed genes and metabolites in endocrine resistant breast cancer cells. Decreased levels of EGR1 in ER+ breast cancer cells and human tumors correlated with decreased sensitivity to antiestrogens. However, sustained inhibition of EGR1 with siRNA or tolfenamic acid (TOLE) suppressed the growth of endocrine resistant breast cancer cells and interacted synergistically with both 4-hydroxytamoxifen (hereafter referred to as TAM), major active metabolite of tamoxifen, and ICI in inhibiting cell proliferation. EGR1 inhibition also disrupted fatty acid metabolism in endocrine resistant cells. Overall, our gene-metabolite integrated model suggests a novel role for EGR1 in regulating cellular metabolism in endocrine resistant breast cancer.

## RESULTS

### Analysis and integration of transcriptomics and metabolomics data from endocrine sensitive and resistant breast cancer cell lines

**Figure 1:**
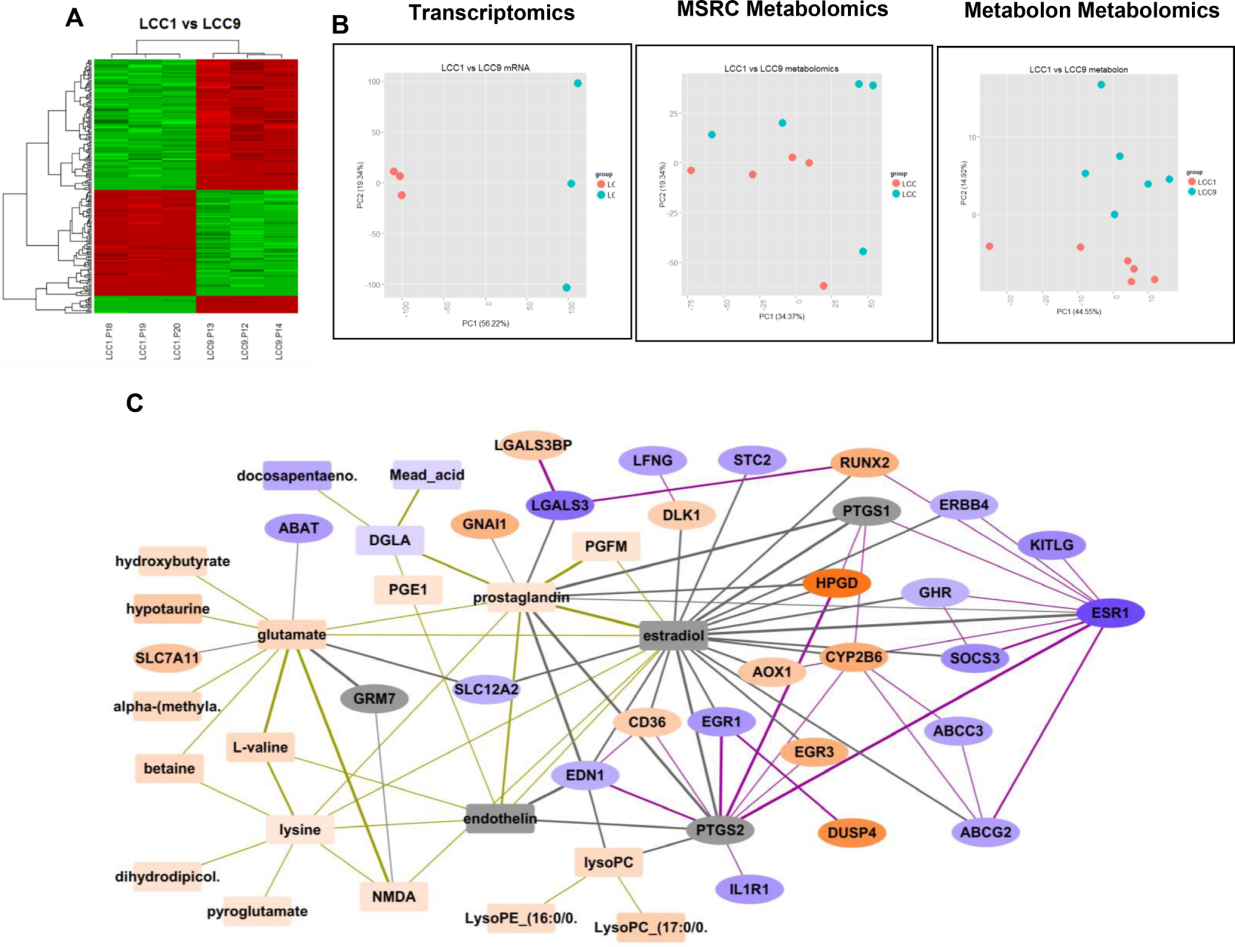
Analysis and integration of gene and metabolites in LCC1 (sensitive) and LCC9 (resistant) ER+ breast cancer cells (**A**) Heatmap: R package Limma was used for microarray analysis of LCC1 versus LCC9 data; significantly different genes were selected (*q*-value < 0.1, fold change, FC > 2) to plot the heatmap; there were 3-biological replicates. (**B**) Principal component analysis: PCA analysis performed for the transcriptomics and metabolomics datasets - MSRC and Metabolon. (**C**) Integration of differentially expressed genes and putative metabolites comparing LCC9 (resistant) and LCC1 (sensitive) cells. Metabolites are shown as rectangular nodes, and genes as ellipses. *Orange* nodes are over-expressed; *blue* nodes are under-expressed; darker color represents a higher fold change (FC). Lowest log2 FC = –5.66, highest log2 FC = 5.89. *Grey* nodes are those added into the network based on prediction by STITCH. Edge thickness increases with the confidence of the connection as predicted by STITCH. Gene-metabolite connections are shown in *grey* lines, gene-gene connections are shows as *purple* lines, and metabolite-metabolite connections are shown as gold lines. EGR1 is significantly decreased in LCC9 cells (log2 FC= –2.33).

Comparing LCC1 and LCC9 cell lines yielded 4,010 unique differentially expressed genes (DEGs), 46 unique m/z values for metabolites from the Metabolomics Shared Resources Core (MSRC) analysis conducted at Georgetown University Medical Center and 12 identified metabolites from the Metabolon analysis at an FDR threshold of 0.01 for the first two analyses, respectively; 0.05 for the third analysis. (Figure 1A and 1B). A heatmap was generated using all differentially expressed genes. Data visualization by principal component analysis (PCA) analysis was performed for both the MSRC and Metabolon metabolome analyses. While there was inherent noise, LCC1 and LCC9 cells were clearly separated in both plots. Figure 1C shows the integrated network of differentially expressed genes and putative metabolites in LCC9 cells (endocrine resistant) using 300 DEGs, 46 unique m/z values for metabolites from the MSRC analysis and 11 identified metabolites from the Metabolon analysis. The genes and metabolites present in the integrated network are also presented in Table 1. Based on our metabolomics analysis, glutamate and prostaglandin levels were significantly higher in LCC9 compared with LCC1 cells (log2 FC=1.522, *p*-value = 0.00011, *q*-value = 0.00574; respectively log2 FC=1.047, *p*-value = 0.00019, *q*-value = 0.00759). Estradiol and endothelin along with three genes (PTGS1, PTGS2 and GRM7) were added to the network based on predictions from the STITCH database.

**Table 1:** Genes and metabolites from integrated network (Figure 1C)

Name	Expanded Name	Type	Probe/Metabolite ID	log2 fold-change	*p*-value	*q*-value
ESR1	ESR1	gene	205225_at	–4.036	2.36E-12	6.91E-09
LGALS3	LGALS3	gene	208949_s_at	–3.232	4.4E-11	3.55E-08
KITLG	KITLG	gene	211124_s_at	–2.666	5.47E-08	4.43E-06
SOCS3	SOCS3	gene	227697_at	–2.575	5.68E-10	1.96E-07
EGR1	EGR1	gene	201694_s_at	–2.333	6.25E-08	4.88E-06
IL1R1	IL1R1	gene	202948_at	–2.331	4.10E-08	3.71E-06
ABAT	ABAT	gene	209459_s_at	–2.219	2.44E-08	2.53E-06
STC2	STC2	gene	203438_at	–2.176	3.24E-10	1.29E-07
ABCG2	ABCG2	gene	209735_at	–2.133	7.61E-10	2.34E-07
LFNG	LFNG	gene	228762_at	–2.121	1.67E-09	4.12E-07
ABCC3	ABCC3	gene	208161_s_at	–2.061	4.81E-09	8.16E-07
ERBB4	ERBB4	gene	214053_at	–1.884	6.01E-09	9.71E-07
EDN1	EDN1	gene	218995_s_at	–1.823	2.36E-09	5.31E-07
SLC12A2	SLC12A2	gene	204404_at	–1.779	3.16E-09	6.38E-07
GHR	GHR	gene	205498_at	–1.734	2.62E-08	2.68E-06
DLK1	DLK1	gene	209560_s_at	1.829	3.2E-09	6.41E-07
CD36	CD36	gene	206488_s_at	1.906	4.86E-08	4.15E-06
LGALS3BP	LGALS3BP	gene	200923_at	2.065	3.38E-09	6.60E-07
AOX1	AOX1	gene	205083_at	2.14	3.54E-10	1.38E-07
SLC7A11	SLC7A11	gene	209921_at	2.231	1.77E-09	4.23E-07
GNAI1	GNAI1	gene	227692_at	2.866	2.59E-10	1.19E-07
EGR3	EGR3	gene	206115_at	2.975	1.21E-08	1.55E-06
RUNX2	RUNX2	gene	232231_at	3.02	4.77E-09	8.15E-07
CYP2B6	CYP2B6	gene	206754_s_at	3.161	1.2E-10	6.69E-08
DUSP4	DUSP4	gene	204014_at	4.272	4.56E-11	3.56E-08
HPGD	HPGD	gene	203913_s_at	5.252	2.16E-10	1.08E-07
GRM7	GRM7	gene	Added to the network			
PTGS1	PTGS1	gene				
PTGS2	PTGS2	gene				
docosapentaeno.	Docosapentaenoic acid (22N-6)	metabolite	HMDB01976	–1.911	0.00001	0.00141
DGLA	8;11;14-Eicosatrienoic acid	metabolite	HMDB02925	–0.938	0.00024	0.00859
Mead_acid	5;8;11-Eicosatrienoic acid	metabolite	HMDB10378	–0.938	0.00024	0.00859
lysine	Lysine	metabolite	HMDB00182	0.872	0.00010	0.00521
pyroglutamate	Pyroglutamic acid	metabolite	HMDB00267	1	0.00012	0.00586
PGE1	Prostaglandin E1	metabolite	HMDB01442	1.047	0.00019	0.00759
PGFM	3,14-dihydro-15-keto PGF2a	metabolite	HMDB04685	1.047	0.00019	0.00759
prostaglandin	Prostaglandin D1	metabolite	HMDB05102	1.047	0.00019	0.00759
NMDA	N-Methyl-D-Aspartic acid	metabolite	HMDB02393	1.094	0.00006	0.00421
dihydrodipicol.	L-2;3-Dihydrodipicolinate	metabolite	HMDB12247	1.094	0.00006	0.00421
LysoPE_(16:0/0.	LysoPE(16:0/0:0)	metabolite	HMDB11503	1.294	0.00017	0.00712
lysoPC	LysoPC (17:0/0.	metabolite	HMDB12108	1.365	0.00002	0.00248
L-valine	L-valine	metabolite	HMDB00883	1.438	0.00013	0.00612
alpha-(methyla.	alpha-(methylamino)isobutyric acid	metabolite	HMDB02141	1.438	0.00005	0.00399
betaine	betaine	metabolite	HMDB00043	1.438	0.00005	0.00399
glutamate	glutamate	metabolite	HMDB03339	1.522	0.00011	0.00574
LysoPC_(17:0/0.	LysoPC(17:0)	metabolite	HMDB12108	1.795	0.00002	0.00248
hydroxybutyrate^*^	hydroxybutyrate	metabolite	HMDB00710	1.297	0.00079	0.03740
hypotaurine^*^	Hypotaurine	metabolite	HMDB00965	2.017	0.00001	0.00132
endothelin	Endothelin	metabolite	Added to the network			
estradiol	Estradiol	metabolite				

The table shows the genes and metabolites from Figure 1C. The metabolite names are putative metabolites, so they could be one of the many annotations obtained. The metabolites marked with ^*^ were from Metabolon and experimentally validated.

Results from the pathway analyses are presented in Table 2. Table 2A represents results using the significant metabolites, Table 2B using the top 300 genes, and Table 2C using both metabolites and genes. In particular, we noted the D-glutamine and D-glutamate metabolism pathway (Table 2A, *p*-value = 0.001, *q*-value = 0.052), several key signaling pathways (Table 2B, and prostaglandin synthesis and regulation (Table 2C, *p*-value < 0.001, *q*-value = 0.002 when combining genes and metabolites).

**Table 2A:** Pathway analysis of significant metabolites (from MSRC and Metabolon) performed using MetaboAnalyst (http://www.metaboanalyst.ca/), showing pathways with *p*-value < 0.05

Name of pathway	Number of significant metabolites in pathway / Number of metabolites in pathway	*p*-value	*q*-value
Arachidonic acid metabolism	7/62	< 0.001	0.020
D-Glutamine and D-glutamate metabolism	3/11	0.001	0.052
Lysine degradation	5/47	0.003	0.073
Arginine and proline metabolism	6/77	0.005	0.101
Sphingolipid metabolism	3/25	0.015	0.237
Glycerophospholipid metabolism	3/39	0.048	0.640

**Table 2B:** Pathway analysis of top 300 genes (according to *q*-value) using Reactome (www.reactome.org), showing pathways with *p*-value < 0.05

Name of pathway	Number of top genes in pathway/ Number of genes in pathway	*p*-value	*q*-value
Translocation of ZAP-70 to Immunological synapse	16/39	< 1.00E-10	< 1.00E-10
Phosphorylation of CD3 and TCR zeta chains	16/44	< 1.00E-10	< 1.00E-10
PD-1 signaling	16/45	< 1.00E-10	< 1.00E-10
Generation of second messenger molecules	16/57	< 1.00E-10	< 1.00E-10
Co-stimulation by the CD28 family	16/96	< 0.001	< 0.001
MHC class II antigen presentation	18/141	< 0.001	< 0.001
Downstream TCR signaling	16/123	< 0.001	< 0.001
Cytokine Signaling in Immune system	41/747	< 0.001	< 0.001
Interferon Signaling	24/291	< 0.001	< 0.001
Interferon gamma signaling	18/176	< 0.001	< 0.001
TCR signaling	16/145	< 0.001	< 0.001
ERBB2 Activates PTK6 Signaling	4/18	< 0.001	0.026
ERBB2 Regulates Cell Motility	4/19	< 0.001	0.029
Interleukin-19, 20, 22, 24	3/9	< 0.001	0.035
Downregulation of ERBB4 signaling	3/10	0.001	0.045
SHC1 events in ERBB2 signaling	4/25	0.002	0.064
Nuclear signaling by ERBB4	5/44	0.002	0.077
Signaling by PTK6	6/80	0.007	0.205
GRB2 events in ERBB2 signaling	3/20	0.008	0.245
Interferon alpha/beta signaling	8/140	0.009	0.252
PTK6 Activates STAT3	2/7	0.009	0.253
PI3K events in ERBB2 signaling	3/22	0.011	0.273
Termination of O-glycan biosynthesis	3/28	0.021	0.450
Growth hormone receptor signaling	3/29	0.022	0.450
Signaling by ERBB2	4/54	0.026	0.450
Activation of anterior HOX genes in hindbrain development during early embryogenesis	6/113	0.030	0.450
Activation of HOX genes during differentiation	6/113	0.030	0.450
Signaling by Interleukins	15/425	0.031	0.450
Constitutive Signaling by Aberrant PI3K in Cancer	5/85	0.032	0.450
Adaptive Immune System	31/1075	0.035	0.450
Immune System	52/1984	0.035	0.450
RA biosynthesis pathway	3/39	0.047	0.450
NCAM signaling for neurite out-growth	11/300	0.047	0.450
ABC-family proteins mediated transport	4/66	0.048	0.450

**Table 2C:** Pathway analysis of top 300 genes (according to *q*-value) and significant metabolites using http://impala.molgen.mpg.de/

Name of pathway	Source of pathway	Pathway analysis for genes			Pathway analysis for metabolites			Pathway analysis for genes and metabolites	
		Number of top genes in pathway/ Number of genes in pathway	*p*-value	*q*-value	Number of significant metabolites in pathway/ Number of metabolites in pathway	*p*-value	*q*-value	*p*-value	*q*-value
Prostaglandin Synthesis and Regulation	Wikipathways	4/28	< 0.001	0.457	2/9	0.016	0.687	< 0.001	0.002
ABC-family proteins mediated transport	Reactome	4/36	< 0.001	0.457	2/10	0.019	0.746	< 0.001	0.006
Synthesis of Prostaglandins (PG) and Thromboxanes (TX)	Reactome	3/15	< 0.001	0.457	3/34	0.038	1	< 0.001	0.009
Arachidonic acid metabolism	Reactome	3/54	0.029	1	7/78	0.001	0.096	< 0.001	0.011
Transmembrane transport of small molecules	Reactome	15/594	0.006	0.63	9/178	0.014	0.618	< 0.001	0.024
Transport of inorganic cations/anions and amino acids/oligopeptides	Reactome	5/94	0.006	0.63	4/46	0.017	0.73	0.001	0.027
GABA synthesis, release, reuptake and degradation	Reactome	2/20	0.025	1	2/15	0.042	1	0.008	0.180

### Decreased EGR1 expression correlates with decreased responsiveness to antiestrogens in human breast tumors

**Figure 2:**
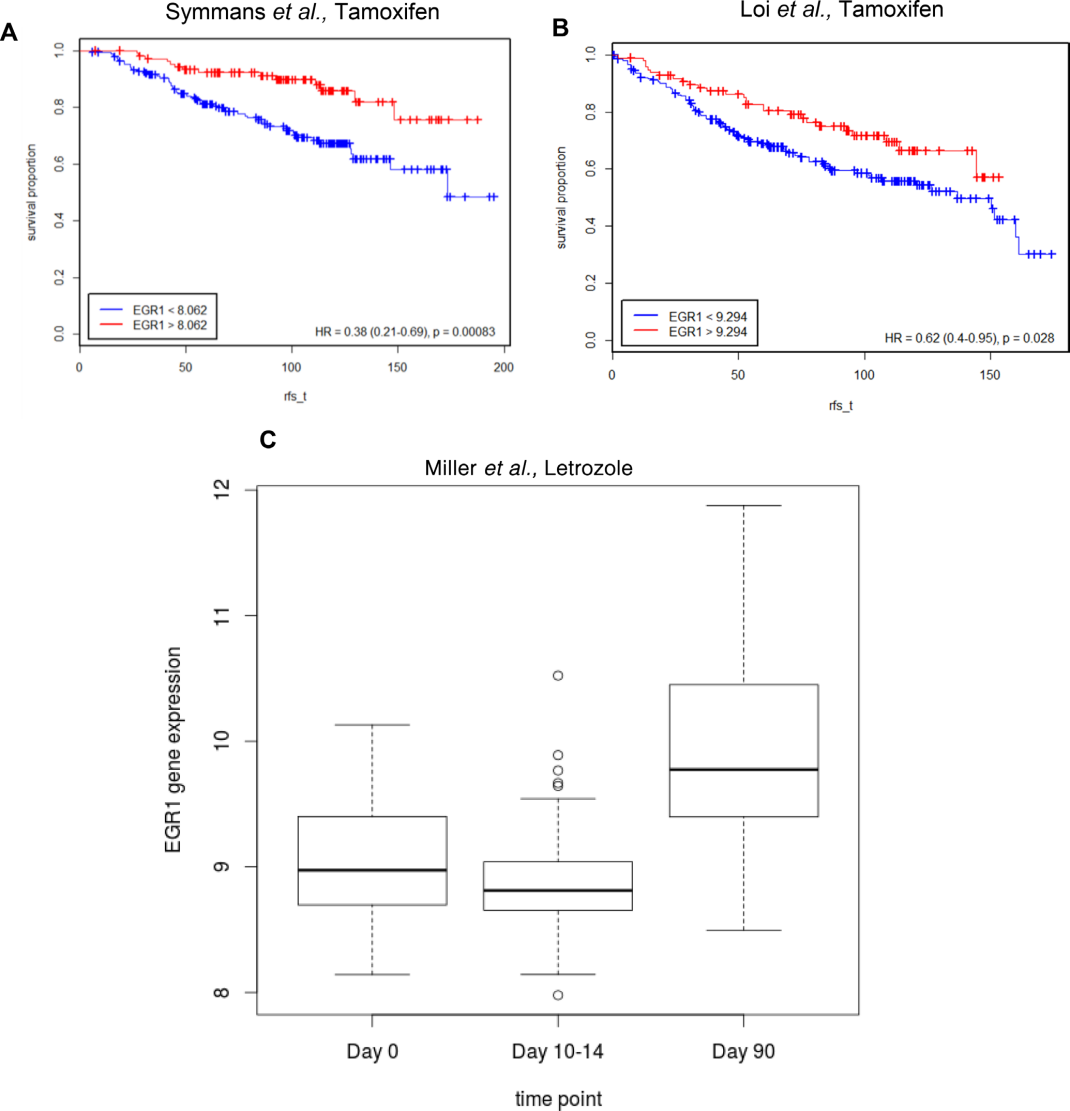
Lower EGR1 levels correlate with lower survival in ER+ breast cancer patients treated with endocrine therapy (**A**) and (**B**) Kaplan-Meier plots were generated using the Symmans *et al.* and Loi *et al.* datasets to estimate the number of patients living over time post endocrine treatment (Tamoxifen) with indicated levels of EGR1 expression in their breast tumors; rfs_t (recurrence free survival time) (**C**) Pre-treatment vs. 90 days post-treatment (Letrozole) comparisons show significantly increased levels of EGR1 expression (*p <* 0.0001) only in the responder group.

To determine whether EGR1 expression was associated with disease free survival, we used publicly available gene expression datasets (see Methods; Figure 2; Table 3) for ER+ human breast tumors treated with endocrine therapy (adjuvant tamoxifen or AI as the only systemic therapy). Kaplan-Meier estimates of relapse-free survival over time (rfs_t) showed that high EGR1 gene expression levels were significantly correlated with favorable prognosis in at least two different datasets where breast tumors were treated with tamoxifen: in Symmans *et al.*, GSE17705 [HR=0.38 (0.21–0.69); *p =* 0.00083] [19] and Loi *et al.,* GSE6532 (ER+ samples on GPL96 platform) [HR=0.62(0.4–0.95); *p =* 0.028] [20] (Figure 2A and 2B). Furthermore, in Miller *et al.,* GSE20181 [21], pre-treatment versus 90 days post-treatment comparisons for treatment with the aromatase inhibitor Letrozole showed significantly increased levels of EGR1 expression (*p <* 0.0001) only in the responder group (Figure 2C).

**Table 3:** Gene expression public dataset for ER+ breast cancer used for correlating EGR1 expression and endocrine response

Dataset	Treatment	Duration	Sample_Size
Symmans *et al.*	Tamoxifen	5 years	298
Loi *et al.*	Tamoxifen	N/A	181
Miller *et al.*	Letrozole	0,10-14,90 day time-point	36 in each time-point

### EGR1 regulates cell proliferation in both endocrine sensitive and resistant breast cancer cell lines

**Figure 3:**
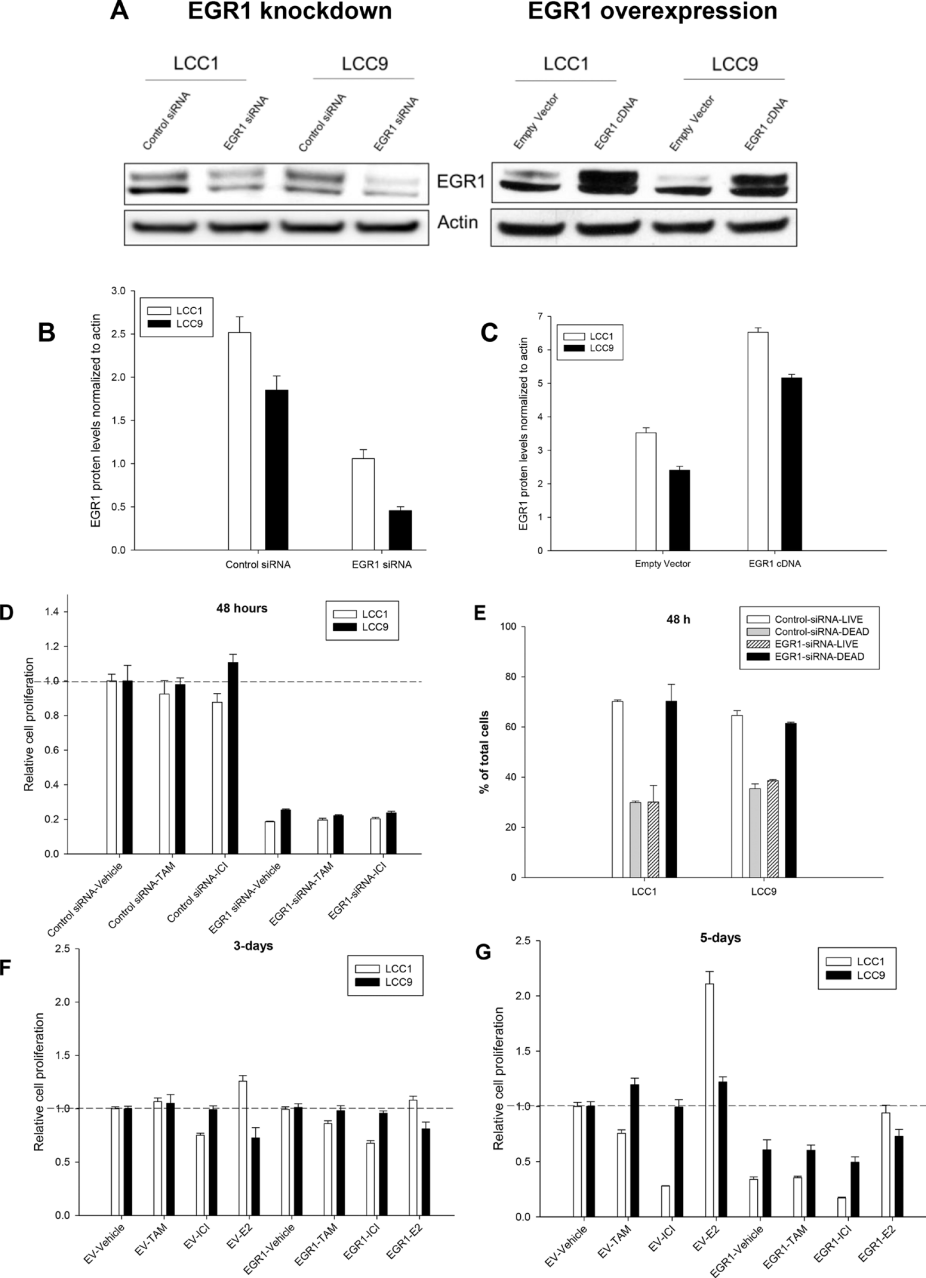
EGR1 expression regulate cell proliferation and viability in both endocrine sensitive and resistant ER+ breast cancer cells (**A**) Western blot of LCC1 and LCC9 cells showing the effect of EGR1 knockdown (EGR1-siRNA) and its respective control (EGR1-control-siRNA) or EGR1 overexpression (EGR1) or its respective control, empty vector (EV). Cells were transfected with siRNA or cDNA plasmid for 72 h. EGR1 protein appeared as a doublet, perhaps due to phosphorylation. Actin was used as a loading control. (**B**–**C**) Quantification of EGR1 protein (normalized to actin) following transfection with EGR1-siRNA compared with control siRNA in LCC1 and LCC9 cells show 2.5- and 3.8-fold reduction, respectively, (B) EGR1 protein in LCC1 and LCC9 cells show 1.4- and 2-fold increase, respectively, with EGR1-cDNA compared with EV, (**C**, **D**) EGR1 knockdown in both LCC1 and LCC9 cells significantly decreased cell proliferation at 48 h regardless of TAM or ICI treatment (ANOVA, *p <* 0.001). (**E**) EGR1 knockdown significantly decreased cell viability in both LCC1 and LCC9 cells (ANOVA, *p <* 0.01; ^*^p < 0.01 for cell death in EGR1-siRNA versus control-siRNA for respective cells lines) at 48 h. (**F**) and (**G**) EGR1 overexpression for 48 h followed by treatment with TAM or ICI for 3-days or 5-days, respectively. While EGR1 overexpression did not change cell proliferation of either LCC1 or LCC9 cell under control or treatment conditions at 3-days, at 5-days, EGR1 transfected LCC1 and LCC9 cells showed significant decrease in cell proliferation compared with respective cells transfected with EV. At 5-day transfection with EGR1 combined with E2 treatment showed a significant decrease in E2 response compared to EV control (ANOVA, *p <* 0.05).

To elucidate the role of EGR1 in endocrine responsiveness, we inhibited (RNAi) or overexpressed the EGR1 cDNA in LCC1 and LCC9 cells, followed by treatment with vehicle (ethanol), TAM, ICI or 17β-estradiol (E2). Figure 3A and 3B shows Western blot data that confirm the EGR1 protein levels with knockdown (EGR1-siRNA) or overexpression (EGR1-cDNA). Figure 3C and 3D show graphs of EGR1 protein levels normalized to actin protein levels from three independent experiments where LCC1 and LCC9 cells were transfected with either EGR1 siRNA, cDNA, or their respective controls. Under control conditions (control siRNA or empty vector/EV), the EGR1 protein levels were 1.25-fold higher in LCC1 cells compared with that in LCC9 cells. Thus, we confirmed the model prediction in Figure 1C that endogenous EGR1 expression is higher in LCC1 cells and lower in LCC9 cells. In EGR1 siRNA transfected cell, EGR1 protein levels decreased 2.5- and 3.8-fold in LCC1 and LCC9 cells, respectively, compared with control siRNA transfected cells. In EGR1 cDNA transfected cell, EGR1 protein levels increased 1.4-fold and 2.5-fold in LCC1 and LCC9 cells, respectively, compared to that in empty vector (EV) transfected cells. Independent of antiestrogen treatment, transfection with EGR1-siRNA significantly reduced cell proliferation in both cell lines within 48 h compared with control-siRNA (Figure 3D). To determine whether EGR1 siRNA changed cell viability, we studied changes in apoptosis and necrosis in LCC1 and LCC9 cells transfected with either control or EGR1 siRNA for 48 h. Figure 3E shows significant decrease in cell viability in both LCC1 and LCC9 cells transfected with control siRNA compared with EGR1 siRNA. Transfection with EGR1-cDNA did not initially affect LCC1 or LCC9 proliferation (Figure 3F). However, 5-days post-transfection, EGR1-cDNA transfected LCC1 and LCC9 cells each exhibited a significant decrease in proliferation compared with their respective EV-transfected controls (Figure 3G). LCC1 cells, at 5-days post-transfection with EGR1-cDNA and treated with ICI, showed a modestly additive growth inhibition relative to EV control cells. At 5-days post-transfection with EGR1-cDNA and E2 treatment, LCC1 cells showed a significant decrease in E2 response compared to their EV controls. Thus, some basal level of EGR1 protein expression may be essential for the survival of both endocrine sensitive and resistant cells, whereas changes beyond this base level in sensitive cells determines their responsiveness to E2.

### EGR1 regulates fatty acid metabolism in endocrine resistant breast cancer cells

**Figure 4:**
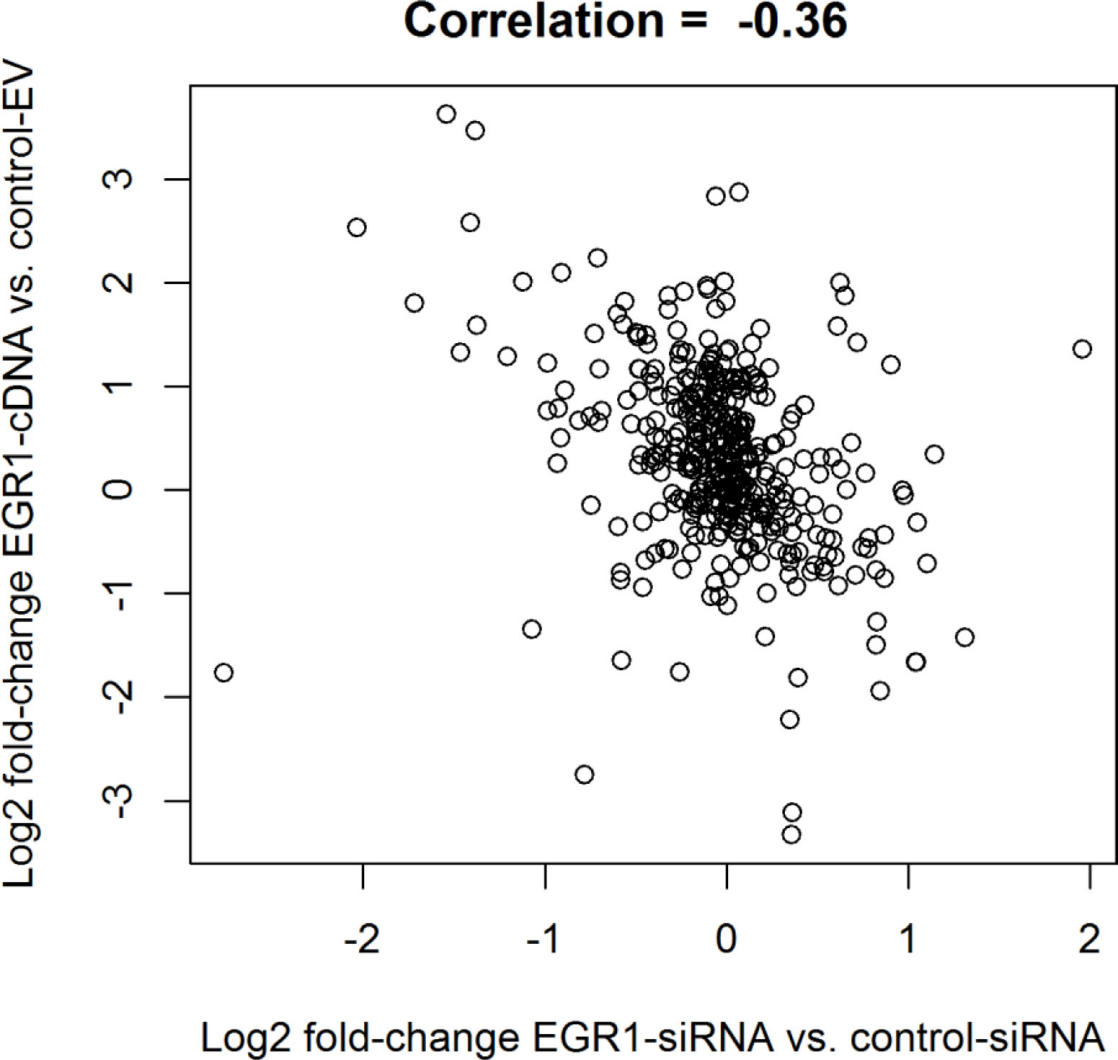
EGR1 knockdown in endocrine resistant cells disrupt fatty acid metabolism pathway Correlation between estimated log2 fold changes for the EGR1 knockdown experiment (siEGR1 vs. siCtrlEGR1) and the estimated log2 fold changes for the EGR1 siRNA experiment (EGR1 cDNA vs. EV EGR1). The negative correlation indicates agreement a global agreement between the two approaches, as the direction of change is expected to be different when comparing the knockdown to the overexpression experiments.

To determine the role of EGR1 in affecting cell metabolism in LCC9 cells, we transfected cells with either the EGR1-siRNA or control siRNA for 48 h, or with the EGR1 cDNA (EGR1-cDNA) and the respective EV controls. Five biological replicates were used for each group. Metabolomics analysis was performed by Metabolon. Data analysis followed the same steps as for the LCC1/LCC9 comparison. Negative correlation between the fold-changes from the two experiments indicated good global agreement between the knockdown and overexpression approaches (Figure 4). Applying an FDR cut off = 0.1 yielded 18 metabolites for the siRNA experiment; 15 of these metabolites had HMDB IDs (Table 4). Pathway analysis of these 15 metabolites was done using MetaboAnalyst [22] and IMPaLA: Integrated Molecular Pathway Level Analysis [23] (Table 5). No metabolites reached statistical significance for the EGR1 overexpression analysis. Several metabolites that were significantly regulated following EGR1 knockdown including 7-hydroxycholesterol and acetyl CoA, had fold-changes in the opposite direction when EGR1 was overexpressed.

**Table 4:** Metabolites that were significantly altered with EGR1-siRNA knockdown in LCC9 cells, with *q*-value < 0.1 with EGR1-siRNA versus EGR1-control siRNA in LCC9 cells

Name	HDMB ID	KEGG ID	Results from siRNA experiment			Results from cDNA experiment		
			log2 fold-change	*p*-value	*q*-value	log2 fold-change	*p*-value	*q*-value
stearoyl-arachidonoyl-glycerophosphoinositol (1)			1.818	< 0.001	0.097	–0.006	0.988	0.999
1-arachidonoylglycerophosphoinositol	HMDB61690		2.829	< 0.001	0.097	–0.060	0.865	0.999
7-hydroxycholesterol (alpha or beta)			–3.116	< 0.001	0.097	0.361	0.392	0.999
desmosterol	HMDB02719	C01802	1.037	< 0.001	0.097	–0.399	0.121	0.999
N-acetylglucosamine	HMDB00215	C00140	1.108	0.002	0.097	–0.418	0.216	0.999
5-dodecenoate (12:1n7)	HMDB00529		1.329	0.002	0.097	–0.220	0.522	0.999
N-palmitoyl-sphingosine	HMDB04949		1.415	0.002	0.097	0.141	0.648	0.999
acetyl CoA	HMDB01206	C00024	–3.331	0.002	0.097	0.358	0.750	0.999
dihomo-linolenate (20:3n3 or n6)	HMDB02925	C03242	1.080	0.002	0.097	0.086	0.730	0.999
linoleate (18:2n6)	HMDB00673	C01595	1.354	0.003	0.097	0.016	0.959	0.999
erucate (22:1n9)	HMDB02068	C08316	1.172	0.003	0.097	0.234	0.454	0.999
1-oleoylglycerophosphoserine			1.107	0.003	0.097	–0.062	0.893	0.999
1-myristoylglycerol (1-monomyristin)	HMDB11561	C01885	1.937	0.003	0.100	–0.105	0.775	0.999
nicotinamide ribonucleotide (NMN)	HMDB00229	C00455	1.076	0.004	0.100	0.170	0.642	0.999
phosphopantetheine	HMDB01416	C01134	2.875	0.004	0.100	0.070	0.952	0.999
caprate (10:0)	HMDB00511	C01571	1.152	0.004	0.100	–0.122	0.693	0.999
arachidonate (20:4n6)	HMDB01043	C00219	1.019	0.004	0.100	0.176	0.493	0.999
uridine 5’-diphosphate (UDP)	HMDB00295	C00015	–1.032	0.004	0.100	–0.040	0.945	0.999

**Table 5:** Pathway analysis on significant metabolites with EGR1-siRNA versus EGR1-control siRNA in LCC9 cells

Name of pathway	Source of pathway	Number of significant metabolites in pathway/ Number of metabolites in pathway	*p*-value	*q*-value
Biosynthesis of unsaturated fatty acids - Homo sapiens (human)	KEGG	4/32	< 0.001	0.042
Linoleic acid metabolism - Homo sapiens (human)	KEGG	3/19	< 0.001	0.042
Signal Transduction	Reactome	6/169	< 0.001	0.042
Heparan sulfate/heparin (HS-GAG) metabolism	Reactome	3/21	< 0.001	0.042
Regulation of lipid metabolism by Peroxisome proliferator-activated receptor alpha (PPARalpha)	Reactome	2/5	< 0.001	0.042
Activation of Gene Expression by SREBP (SREBF)	Wikipathways	2/5	< 0.001	0.042
YAP1- and WWTR1 (TAZ)-stimulated gene expression	Wikipathways	2/5	< 0.001	0.042
Glycosaminoglycan metabolism	Wikipathways	3/27	< 0.001	0.042
Leishmaniasis - Homo sapiens (human)	KEGG	2/6	< 0.001	0.042
Circadian Clock	Wikipathways	2/6	< 0.001	0.042
triacylglycerol degradation	HumanCyc	3/29	< 0.001	0.042
Defective SLC26A2 causes chondrodysplasias	Reactome	3/29	< 0.001	0.042
Defective PAPSS2 causes SEMD-PA	Reactome	3/29	< 0.001	0.042
Defective B4GALT7 causes EDS_ progeroid type	Reactome	3/29	< 0.001	0.042
Defective B3GAT3 causes JDSSDHD	Reactome	3/29	< 0.001	0.042
Defective CHSY1 causes TPBS	Reactome	3/29	< 0.001	0.042
Defective CHST3 causes SEDCJD	Reactome	3/29	< 0.001	0.042
Defective CHST14 causes EDS_ musculocontractural type	Reactome	3/29	< 0.001	0.042
Defective B4GALT1 causes B4GALT1-CDG (CDG-2d)	Reactome	3/29	< 0.001	0.042
Defective CHST6 causes MCDC1	Reactome	3/29	< 0.001	0.042
Diseases associated with glycosaminoglycan metabolism	Reactome	3/29	< 0.001	0.042
Glycosaminoglycan metabolism	Reactome	3/29	< 0.001	0.042
Defective EXT2 causes exostoses 2	Reactome	3/29	< 0.001	0.042
Defective EXT1 causes exostoses 1_ TRPS2 and CHDS	Reactome	3/29	< 0.001	0.042
MPS IX - Natowicz syndrome	Reactome	3/29	< 0.001	0.042
MPS I - Hurler syndrome	Reactome	3/29	< 0.001	0.042
MPS II - Hunter syndrome	Reactome	3/29	< 0.001	0.042
MPS IIIA - Sanfilippo syndrome A	Reactome	3/29	< 0.001	0.042
MPS IIIB - Sanfilippo syndrome B	Reactome	3/29	< 0.001	0.042
MPS IIIC - Sanfilippo syndrome C	Reactome	3/29	< 0.001	0.042
MPS IIID - Sanfilippo syndrome D	Reactome	3/29	< 0.001	0.042
MPS IV - Morquio syndrome A	Reactome	3/29	< 0.001	0.042
MPS IV - Morquio syndrome B	Reactome	3/29	< 0.001	0.042
MPS VI - Maroteaux-Lamy syndrome	Reactome	3/29	< 0.001	0.042
MPS VII - Sly syndrome	Reactome	3/29	< 0.001	0.042
Mucopolysaccharidoses	Reactome	3/29	< 0.001	0.042
phospholipases	HumanCyc	3/30	< 0.001	0.044
sphingomyelin metabolism/ceramide salvage	HumanCyc	3/30	< 0.001	0.044
sphingosine and sphingosine-1-phosphate metabolism	HumanCyc	3/36	< 0.001	0.063
the visual cycle I (vertebrates)	HumanCyc	3/36	< 0.001	0.063
Transport of fatty acids	Reactome	2/9	< 0.001	0.070
Transcriptional Regulation of White Adipocyte Differentiation	Wikipathways	2/9	< 0.001	0.070
Regulation of Lipid Metabolism by Peroxisome proliferator-activated receptor alpha (PPARalpha)	Wikipathways	2/9	< 0.001	0.070

The pathway analysis was performed using tool http://impala.molgen.mpg.de/ . The pathways enriched at *q*-value < 0.1 are shown.

Results from the siRNA and cDNA experiments suggest that disrupted EGR1 expression may have a subtle impact on the metabolic profile of LCC9 breast cancer cells. However, since cell proliferation was significantly reduced in with EGR1-siRNA (at 48 h; Figure 3B) and with EGR1-cDNA (at 5-days; Figure 3D), these seemingly subtle metabolic changes at 48 h post-transfection likely underestimate their ability to affect cell phenotype.

Pathway analysis using the significant metabolites from the siRNA experiment is presented in Table 5. Two of the pathways implicate fatty acid metabolism (biosynthesis of unsaturated fatty acids, transport of fatty acids). Fatty acids are a critical source of energy for mitochondrial oxidation and cellular ATP generation. Silencing of EGR1 was accompanied by high levels of glycerol and multiple monoacylglycerols such as 1-myristoylglycerol that may reflect an increase in complex lipid hydrolysis. Consequently, long chain fatty acids such as palmitoleate and medium chain fatty acids including caprylate and heptanoate were also elevated compared to control-siRNA cells. Fatty acid availability may ultimately alter mitochondrial β-oxidation.

### TOLE down-regulates EGR1 and sensitizes resistant cells to antiestrogens

**Figure 5:**
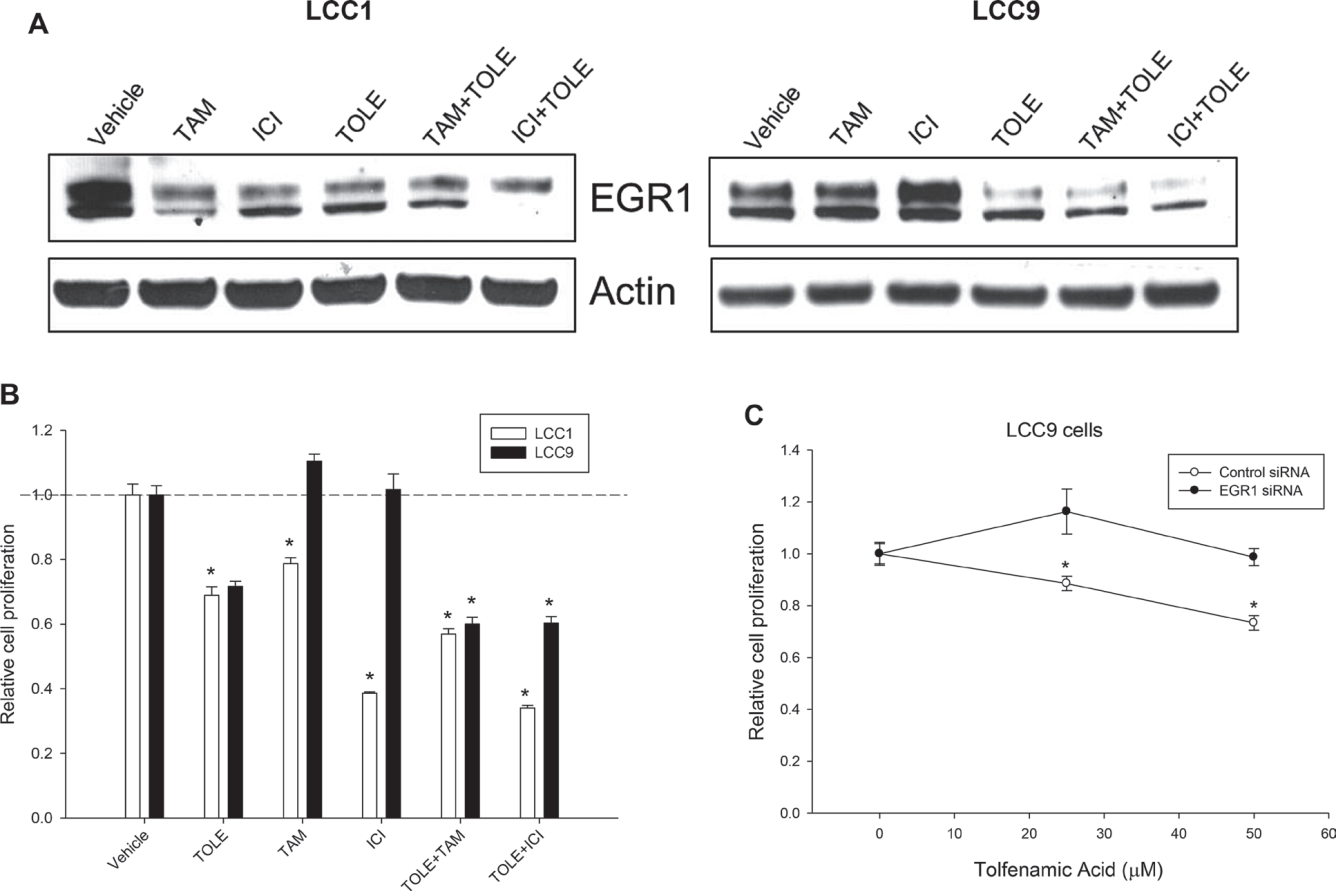
TOLE decreased EGR1 protein in both sensitive and resistant cells and re-sensitize resistant cells to antiestrogens (**A**) Western blot analysis of LCC1 and LCC9 cells, treated with vehicle, TOLE (50 μM), TAM (100 nM) or ICI (100 nM) or the combination for 72 h. In LCC1 cells, TOLE, TAM or ICI treatment decreased EGR1 protein levels. However, in LCC9 cells, antiestrogens increased but TOLE deceased EGR1 protein levels. Actin was used as a loading control. (**B**) Cell proliferation was significantly decreased in both LCC1 and LCC9 with treatment with TOLE at 72 h. Combination of TAM or ICI with TOLE did not show a significant interaction in LCC1 cells. However, cell proliferation was synergistically decreased in LCC9 cells treated with TOLE +TAM (RI = 1.31) or ICI+TOLE (RI = 1.20) within 72 h. ANOVA, *p <* 0.001; *p < 0.001 for specified treatment and cell line compared to vehicle. Dashed line denotes decrease in relative cell proliferation in each cell line with TOLE alone. (**C**) In LCC9 cells, knockdown of EGR1 with siRNA showed significant decrease in cell proliferation with 25 or 50 µM TOLE in LCC9 cells suggesting that TOLE-mediated EGR1 downregulation contributes to TOLE-induced decrease in cell proliferation in LCC9 cells. ANOVA, *p <* 0.05; ^*^p < 0.05 for indicated concentration of TOLE in control-siRNA versus EGR1-siRNA.

Since EGR1 is an essential regulator of cell survival, we tested the effect of TOLE, a nonsteroidal anti-inflammatory drug (NSAID) that induced cell death in an EGR1-dependent manner in colorectal cancer cells [24]. Western blot analysis of whole cell lysates from LCC1 cells (Figure 5A, left panel) show decreased levels of EGR1 at 48 h following treatment with 100 nM TAM or ICI, or 50 µM TOLE. In LCC9 cells (Figure 5A, right panel), treatment with TAM and ICI increased EGR1 protein levels at 72 h compared with control cells. TOLE treatment decreased EGR1 levels in LCC9 cells. In both LCC1 and LCC9 cells, co-treatment with TOLE and either TAM or ICI decreased EGR1 levels. In LCC1 cells, treatment with 100 nM TAM or ICI alone significantly (*p <* 0.001) inhibited cell number; a combination of 50 µM TOLE and TAM or ICI significantly (*p <* 0.001) decreased cell number compared with the individual treatments but the interaction was not synergistic (Figure 5B). In LCC9 cells, treatment with TAM or ICI had no effect on cell number compared with vehicle, but TOLE treatment alone (*p <* 0.001) or in combination with either TAM (RI = 1.31; *p <* 0.001) or ICI (RI = 1.20; *p <* 0.001) synergistically reduced the number of cells within 72 h, implying a partial restoration of ICI sensitivity. To confirm that inhibition of cell number with TOLE was mediated through EGR1 downregulation, we treated LCC9 cells that were either transfected with control siRNA or EGR1 siRNA followed by treatment with increasing doses of TOLE (0–50 µM). EGR1 knockdown resulted in a significant decrease in TOLE-mediated inhibition in cell number at 25 and 50 µM (*p <* 0.05; Figure 5C). Together, these data indicate that antiestrogen treatment can differentially affect EGR1 levels in endocrine sensitive versus resistant cells. Furthermore, TOLE can downregulate EGR1 levels and re-sensitize endocrine resistant cells to antiestrogens.

## DISCUSSION

Molecular adaptations that lead to drug resistance in cancer cells are largely dependent on cellular context and the nature of the stress signal. To determine the pathways that promote endocrine resistance, we integrated gene expression data with metabolite concentrations studying only those signals that were significantly changed in resistant breast cancer cells (LCC9) compared with sensitive cells (LCC1). The resulting model implicated EGR1 as a gene that is downregulated in endocrine resistant cells and proposed an increased activation of the glutamine and arachidonic pathways (Figure 1C; Tables 2 and 3). Interestingly, pathway analysis of top DEGs showed that a number of pathways associated with immune response were significantly altered in endocrine resistant LCC9 cells. It is still unclear how immune response genes are regulated in ER+ breast cancer cells and tumors. In breast cancer patients, depression in cellular immunity was associated with resistance to endocrine therapy [25]. *In vivo* models of mammary tumors suggest a role of immune-associated genes in antiestrogen resistance [26, 27]. The signaling interactions between cancer cells and the tumor microenvironment remain to be elucidated. In this study, to validate our gene-metabolite integration model, we tested whether alteration of EGR1, which is down-regulated in LCC9 cells [15], changed endocrine responsiveness or survival in resistant cells. We also asked whether gene expression data from human tumors treated with endocrine therapy showed a correlation between higher EGR1 levels and a more favorable prognosis (Figure 2A).

EGR1 levels are high in some prostate cancers [28, 29], Wilm’s tumors [30], and melanoma cells bearing oncogenic B-RAF mutation [31] compared to normal tissue. An array of stress stimuli including radiation, chemotherapy, or hypoxia can alter EGR1 levels and the nature of a response is determined by whether EGR1 transcriptionally up- or down-regulates specific target genes [6, 7, 32]. While the precise role of EGR1 in cell survival remains unclear, disruption of endogenous levels of EGR1 can either inhibit growth or promote tumor progression [33, 34]. In prostate cancer cell lines, EGR1 inhibition decreased cell growth, induced apoptosis [35], and decreased expression of the pro-inflammatory chemokine interleukin 8 (IL8) in a NF-κB-dependent pathway [36]. EGR1 has been implicated in the acquisition of resistance to hormone therapy particularly through its role in the androgen receptor (AR) pathway [37, 38]. In breast cancer, the role of EGR1 remains ambiguous. In an ER+, carcinogen-induced (7,12-dimethylbenz(a) anthracene; DMBA), rat mammary tumor model, EGR1 levels in tumors were reduced relative to normal mammary tissues but then increased with TAM treatment [39]. In ER-negative breast cancer, the EGR1 gene is frequently deleted [13]. Disruption of the ER signaling pathway can affect EGR1 levels in ER+ breast cancer cells and tumors [14, 39]. Interestingly, overexpression of EGR1 dramatically reduced E2-mediated proliferation in these cells. In a model to identify topological and temporal effects of E2 regulatory networks in MCF7 cells, EGR1 was identified as a mediator of some of the late responses to E2 [40]. Thus, EGR1 levels in breast cancer cells may be closely regulated by a functional ER pathway.

A role for EGR1 in regulating cellular metabolic pathways has been reported in several disease models including cancer. Several cellular metabolic pathways were altered with perturbation of EGR1 levels in LCC9 cells (Table 5), particularly molecules associated with fatty acid metabolism. EGR1 and the lipogenic enzyme fatty acid synthase (FASN) are elevated in tissues adjacent to prostate cancer; this relationship is used as a predictive marker of recurrence [41]. FASN activation is required for estrogen-mediated signaling in ER+ breast cancer cells [42] . EGR1 is an immediate-early prostaglandin E2 (PGE2) target gene that can mediate eicosanoid regulation of genes involved in the immune and inflammatory responses [43]. Together, these findings highlight the critical role of EGR1 in fatty acid metabolism.

In LCC9 cells, combining TOLE and antiestrogens synergistically inhibited cell growth (Figure 5B). TOLE can inhibit synthesis of prostaglandins and has been used for treating migraines [44]. In a triple-negative breast cancer xenograft model, treatment with TOLE resulted in a significant reduction in tumor volume over 5 weeks compared to treatment with vehicle alone [45]. TOLE can inhibit cell growth in cancer cells through cyclooxygenase-independent pathways including inhibition of ErbB2 expression [46], activation or ATF3 [47], or induction of NSAID-activated gene-1 (NAG-1) [48] and EGR1 [24]. Contrary to the latter study, in our breast cancer cells, treatment with TOLE inhibited EGR1 protein levels in LCC9 cells and synergized with ICI and TAM (Figure 5B). Thus, cellular context is likely a key determinant of the outcomes of EGR1 action.

Overall, we present a comprehensive model incorporating the differential expression/concentration of genes and metabolites that may interact to determine breast cancer cell fate in the response to select endocrine therapies. We tested our model by further elucidating the role of EGR1 in endocrine sensitive and resistant breast cancer cells. Our data suggest that although EGR1 levels are significantly reduced in endocrine resistant cells, it is an essential driver of cell survival and metabolic pathways such as fatty acid metabolism. Targeting EGR1 with TOLE may be an effective therapeutic strategy in some endocrine resistant breast cancers. Furthermore, EGR1 levels in human breast tumors may be useful as a favorable prognosis marker in ER+ breast cancer.

## MATERIALS AND METHODS

### Cell culture and reagents

LCC1 (sensitive) and LCC9 (ICI resistant and TAM cross-resistant) cells were established as previously described [49–51]. Cells were grown in phenol red–free IMEM (Life Technologies, Grand Island, NY; A10488-01) with 5% charcoal-stripped calf serum (CCS); this media contains 2 mM L-glutamine and ∼12 mM glucose. ICI182,780 (ICI) and 4-hydroxytamoxifen (TAM) were obtained from Tocris Bioscience (Ellisville, MO). Tolfenamic acid (TOLE) was purchased from Selleck (Houston, TX). All cells were authenticated by DNA fingerprinting and tested regularly for *Mycoplasma* infection. All other chemicals were purchased from Sigma-Aldrich.

### Cell proliferation and viability

For determination of cell density, cells were plated in 96-well plates at 5 × 10^3^ cells/well. At 24 h, cells were treated with specified drugs for 48 h (or otherwise indicated). After treatment, media were removed, and plates were stained with a solution containing 0.5% crystal violet and 25% methanol, rinsed, dried overnight, and re-suspended in citrate buffer (0.1 M sodium citrate in 50% ethanol). Intensity of staining, assessed at 570 nm and quantified using a V_Max_ kinetic microplate reader (Molecular Devices Corp., Menlo Park, CA), is directly proportional to cell number [50, 51]. For assessing cell viability and cell death (apoptosis and necrosis), cells were treated for 48 h, and stained with an Annexin V-fluorescein isothiocyanate and propidium iodide, respectively (Trevigen, Gaithersburg, MD).

### Transfections with EGR1 siRNA or cDNA

Cells were plated at 60–80% confluence. EGR1 (10 nM of 3 unique 27mer siRNA duplexes; Origene, Rockville, MD) or their respective control siRNA, were transfected using the RNAiMAX (Invitrogen) transfection reagent. For EGR1 overexpression, EGR1 cDNA (catalog #SC128132) was purchased from Origene and transfected with TransIT-2020 (Mirus). Cells were lysed at 48 h post-transfection and subjected to Western blot analysis or cell number assay as described above. Antiestrogens, 100 nM ICI or TAM, or vehicle (0.02% ethanol) was added to the transfected cells at 24 h and treatment was allowed for the time-points indicated. For 5-day treatments, cells were re-treated with the indicated drugs in fresh cell culture media at day 3.

### Western analyses

Total protein (∼20 μg) was isolated from cells following 72 h treatment or vehicle control (0.02% DMSO or ethanol) for protein analysis as previously described [50, 51]. The following antibodies were used: EGR1 (Cell Signaling, Danvers, MA), and β-actin (Santa Cruz Biotechnology, Santa Cruz, CA).

### Generation and integration of transcriptomic and metabolomic data from LCC1 and LCC9 cells

#### Transcriptome data

We obtained and analyzed gene expression and untargeted metabolomics data from antiestrogen sensitive (LCC1) or antiestrogen resistant (LCC9). Microarray analysis was performed using three biological replicates from LCC1 and three biological replicates from LCC9 using Affymetrix HG U133 Plus 2.0 microarray at our Genomics and Epigenomics Shared Resources. Briefly, total RNA was extracted using the RNeasy kit (Qiagen, Valencia, CA, USA). RNA labeling and hybridization were performed according to the Affymetrix protocol for one-cycle target labeling. For each experiment, fragmented cRNA was hybridized in triplicates to Affymetrix GeneChip HG-U95 arrays (Affymetrix, Santa Clara, CA). Affymetrix data analysis included pre-processing of the probe-level Affymetrix data (CEL files).

### Metabolomics data

Metabolomics analysis was two-part: we sent cell samples to both our in-house MSRC and to Metabolon Inc. MSRC samples were five biological replicates from each of the two groups, each with two technical replicates. Metabolon samples were six biological replicates from each of the two groups. LC-MS was used to analyze the MSRC samples; both LC-MS and GC-MS were used by Metabolon.

For the MSRC protocol, metabolite extraction was performed as described by Sheikh *et al.* [52]. Briefly, the residual pellet was resuspended in 200 μL of solvent A (98% water, 2% ACN and 0.1% formic acid) for Ultra-performance liquid chromatography-electro-spray ionization quadrupole-time-of-flight mass spectrometry (UPLC-ESI-Q-TOFMS) analysis. Mass spectrometry was performed on a Q-TOF Premier (Waters) operating in either negative-ion (ESI-) or positive-ion (ESI+) electro-spray ionization mode with a capillary voltage of 3200 V and a sampling cone voltage of 20 V in negative mode and 35 V in positive mode. The cone gas flow was 25 L/h, and the source temperature was 120°C. Accurate mass was maintained by introduction of LockSpray interface of sulfadimethoxine (311.0814 [M+H]+ or 309.0658 [M-H]−). Data were acquired in centroid mode from 50 to 850 m/z in MS scanning. Centroided and integrated mass spectrometry data from the UPLC-TOFMS was processed to generate a multivariate data matrix using MarkerLynx (Waters).

For Metabolon, samples were prepared using the automated MicroLab STAR^®^ system (Hamilton Company, Reno, NV). A recovery standard was added prior to the first step in the extraction process for quality control (QC) purposes. Proteins were precipitated with methanol to remove protein, dissociate small molecules bound to protein or trapped in the precipitated protein matrix, and to recover chemically diverse metabolites. The resulting extract was divided into five fractions: one for analysis by UPLC-MS/MS with positive ion mode electrospray ionization, one for analysis by UPLC-MS/MS with negative ion mode electrospray ionization, one for LC polar platform, one for analysis by GC-MS, and one sample was reserved for backup. Samples were placed briefly on a TurboVap^®^ (Zymark) to remove the organic solvent. For LC, the samples were stored overnight under nitrogen before preparation for analysis. For GC, each sample was dried under vacuum overnight before preparation for analysis. The LC/MS portion of the platform was based on a Waters ACQUITY ultra-performance liquid chromatography (UPLC) and a Thermo Scientific Q-Exactive high resolution/accurate mass spectrometer interfaced with a heated electrospray ionization (HESI-II) source and Orbitrap mass analyzer operated at 35,000 mass resolution. The MS analysis alternated between MS and data-dependent MS2 scans using dynamic exclusion, and the scan range was from 80–1000 m/z. The samples destined for analysis by GC-MS were were analyzed on a Thermo-Finnigan Trace DSQ fast-scanning single-quadrupole mass spectrometer using electron impact ionization (EI) and operated at unit mass resolving power. The scan range was from 50–750 m/z. Raw data files are archived and extracted as described below. The scope of the Metabolon LIMS system encompasses sample accessioning, sample preparation and instrumental analysis and reporting and advanced data analysis. It has been modified to leverage and interface with the MSRC information extraction and data visualization systems, as well as third party instrumentation and data analysis software.

### Metabolite quantification and data normalization

For the MSRC metabolomics data, peaks were detected and quantified using the estimated area-under-the-curve of the LC/MS signals via the CentWave algorithm [53]. For the Metabolon data, an in-house software was used for detection and integration of peaks [54]. For studies spanning multiple days, a data normalization step was performed to correct variation resulting from instrument inter-day tuning differences. Essentially, each compound was corrected in run-day blocks by registering the medians to equal one (1.00) and normalizing each data point proportionately (termed the ‘block correction’). For studies that did not require more than one day of analysis, no normalization was necessary, other than for data visualization. In certain instances, biochemical data were normalized to an additional factor (such as cell counts, total protein as determined by Bradford assay, osmolality) to account for differences in metabolite levels due to differences in the amount of material present in each sample.

### Data analysis and integration of transcriptomics and metabolomics

Gene expression raw data were processed using the RMA algorithm as implemented in the R affy package [55], followed by a log2-transformation and analysis using a moderated *t*-test via the limma approach implemented in the limma package in Bioconductor [56]. MSRC metabolomics data were processed using the XCMS approach [53, 57, 58]. Internal controls were used to standardize raw values. Intensity values were then log2-transformed and quantile normalized [59], to avoid infinite value produced by 0 expression level during the log2-transform phase, 1e-06 was introduced to replace all 0 in the expression profile and the two technical replicates were averaged. Putative isotopes were identified via the CAMERA package [60] and higher-weight isotopes removed. Finally, results were analyzed using limma along with surrogate variable analysis [61, 62] to remove sources of variability unrelated to the comparison of interest. Metabolon data were processed by Metabolon as described above and log2-transformed, followed by the use of limma. Log2 transformation was performed for both data types to meet the normality assumptions for *t*-tests. All statistical tests were adjusted for multiple testing using the Benjamini-Hochberg approach to control the false discovery rate (FDR). Results are presented as both the *p*-values and the *q*-values (transformed *p*-values used for FDR control).

Metabolites significant at an FDR of < 0.01 and < 0.05 for the MSRC set and the Metabolon set, respectively, were annotated using the HMDB and Metlin databases [63, 64]. Pathway analyses for the genes significant at FDR of < 0.01 were performed using Pathway Studio (http://www.elsevier.com/online-tools/pathway-studio) and Enrichr [65] that allowed us to perform pathway analysis using the KEGG [66] and Reactome [67] databases. Pathway analyses for the top metabolites significant at an FDR of < 0.01 for our MSRC analysis and < 0.05 for the Metabolon analysis were performed using MetaboAnalyst [22] and IMPaLA: Integrated Molecular Pathway Level Analysis [23], respectively.

The top 300 DEGs according to their *q*-value were chosen for integration, along with the metabolites obtained after the filtering described above. Knowledge-based integration of these gene expression and metabolomics results was performed using STITCH [68]. This created a network of genes and metabolites using text mining and database curation sources. Based on the information obtained from these sources, each connection was given a confidence value. For our network, connections with at-least medium confidence were selected [68, 69]. STITCH lists up to 50 articles that support each connection in the network. For each article, the PubMed ID and abstract was provided and the information therein was compared for consistency/relevance to the model solution. STITCH added nodes (genes or metabolites) to the predicted network to indicate an indirect connection between two input nodes. A network was created using the nearest 5 and nearest 10 interactions of the input genes and metabolites. The final network obtained in STITCH was downloaded, and formatted using Cytoscape [70] to overlay fold change values from our experiments and to visualize the network.

For EGR1 inhibition and overexpression experiments, five biological replicates were considered and the analysis was performed by Metabolon. The data analysis followed the same steps as that described above for the LCC1/LCC9 comparison. A relaxed FDR cut off of 0.1 was applied for each experiment. Since transfection with EGR1 siRNA in LCC9 cells resulted in significant reduction in cell proliferation at 48 h, the small metabolic changes at this time-point are likely sufficient to affect cell phenotype. The metabolites showing significant differences between the control group and the inhibition or overexpression group were considered in pathway analyses.

### Estimates of relapse-free survival and EGR1 gene expression levels from public gene expression datasets

Publicly available datasets for gene expression from human ER+ breast cancer tumors that were treated with endocrine therapy were obtained: GSE17705 (Symmans *et al.,* treated with Tamoxifen) [19], GSE6532, ER+ samples on GPL96 platform (Loi *et al.*, treated with Tamoxifen) [20] and GSE20181 (Miller *et al.,* treated with Letrozole) [21]. Kaplan-Meier plots were generated using the Symmans *et al.* and Loi *et al.* datasets to estimate the number of patients living over time post endocrine treatment with indicated levels of EGR1 expression in their breast tumors. The dataset in Miller *et al.* was used to generate box plots to show difference in EGR1 expression in responders. Graphs were generated using tools in the R statistical programming language.

### Statistical analysis for cell proliferation experiments

Statistical analyses were performed using the Sigmastat software package (Jandel Scientific, SPSS, Chicago, IL). Where appropriate, cell growth under different conditions were compared using ANOVA with a *post hoc t*-test for multiple comparisons. Differences were considered significant at *p* ≤ 0.05. Nature of interaction between TOLE, TAM and ICI was defined by measuring the R-index (RI). The RI values were obtained by calculating the expected cell survival (S_exp_; the product of survival obtained with drug A alone and the survival obtained with drug B alone) and dividing S_exp_ by the observed cell survival in the presence of both drugs (S_obs_). S_exp_/S_obs_ > 1.0 indicates a synergistic interaction [71].

## Abbreviations

AI: aromatase inhibitors
DEG: differentially expressed genes
EGR1: early growth response 1
E2: 17β-estradiol
ER: estrogen receptor
EV: empty vector
ICI: ICI182,780
MSRC: Metabolomics Shared Resources Core
PCA: principal component analysis
TAM: 4-hydroxytamoxifen
TOLE: Tolfenamic acid

## References

[1] Clarke R, Skaar TC, Bouker KB, Davis N, Lee YR, Welch JN, Leonessa F (2001). Molecular and pharmacological aspects of antiestrogen resistance. J Steroid Biochem Mol Bio.

[2] Clarke R, Liu MC, Bouker KB, Gu Z, Lee RY, Zhu Y, Skaar TC, Gomez B, O’Brien K, Wang Y, Hilakivi-Clarke LA (2003). Antiestrogen resistance in breast cancer and the role of estrogen receptor signaling. Oncogene.

[3] Miller TW, Balko JM, Ghazoui Z, Dunbier A, Anderson H, Dowsett M, Gonzalez-Angulo AM, Mills GB, Miller WR, Wu H, Shyr Y, Arteaga CL (2011). A gene expression signature from human breast cancer cells with acquired hormone independence identifies MYC as a mediator of antiestrogen resistance. Clin Cancer Res.

[4] Musgrove EA, Sergio CM, Loi S, Inman CK, Anderson LR, Alles MC, Pinese M, Caldon CE, Schutte J, Gardiner-Garden M, Ormandy CJ, McArthur G, Butt AJ (2008). Identification of functional networks of estrogen- and c-Myc-responsive genes and their relationship to response to tamoxifen therapy in breast cancer. PLoS One.

[5] Shajahan AN, Riggins RB, Clarke R (2009). The role of X-box binding protein-1 in tumorigenicity. Drug News Perspect.

[6] Adamson E, De Belle I, Mittal S, Wang Y, Hayakawa J, Korkmaz K, O’Hagan D, McClelland M, Mercola D (2003). Egr1 signaling in prostate cancer. Cancer Biol Ther.

[7] Adamson ED, Mercola D (2002). Egr1 transcription factor: multiple roles in prostate tumor cell growth and survival. Tumour Biol.

[8] Sukhatme VP, Cao XM, Chang LC, Tsai-Morris CH, Stamenkovich D, Ferreira PC, Cohen DR, Edwards SA, Shows TB, Curran T, Le Beau MM, Adamson ED (1988). A zinc finger-encoding gene coregulated with c-fos during growth and differentiation, and after cellular depolarization. Cell.

[9] Svaren J, Sevetson BR, Apel ED, Zimonjic DB, Popescu NC, Milbrandt J (1996). NAB2, a corepressor of NGFI-A (Egr-1) and Krox20, is induced by proliferative and differentiative stimuli. Mol Cell Biol.

[10] Swirnoff AH, Apel ED, Svaren J, Sevetson BR, Zimonjic DB, Popescu NC, Milbrandt J (1998). Nab1, a corepressor of NGFI-A (Egr-1), contains an active transcriptional repression domain. Mol Cell Biol.

[11] Gitenay D, Baron VT (2009). Is EGR1 a potential target for prostate cancer therapy?. Future Oncol.

[12] Russell DL, Doyle KM, Gonzales-Robayna I, Pipaon C, Richards JS (2003). Egr-1 induction in rat granulosa cells by follicle-stimulating hormone and luteinizing hormone: combinatorial regulation by transcription factors cyclic adenosine 3′,5′-monophosphate regulatory element binding protein, serum response factor, sp1, and early growth response factor-1. Mol Endocrinol.

[13] Ronski K, Sanders M, Burleson JA, Moyo V, Benn P, Fang M (2005). Early growth response gene 1 (EGR1) is deleted in estrogen receptor-negative human breast carcinoma. Cancer.

[14] Pratt MA, Satkunaratnam A, Novosad DM (1998). Estrogen activates raf-1 kinase and induces expression of Egr-1 in MCF-7 breast cancer cells. Mol Cell Biochem.

[15] Gu Z, Lee RY, Skaar TC, Bouker KB, Welch JN, Lu J, Liu A, Zhu Y, Davis N, Leonessa F, Brunner N, Wang Y, Clarke R (2002). Association of interferon regulatory factor-1, nucleophosmin, nuclear factor-kappaB, and cyclic AMP response element binding with acquired resistance to Faslodex (ICI 182,780). Cancer Res.

[16] Jeon HM, Lee SY, Ju MK, Kim CH, Park HG, Kang HS (2013). Early growth response 1 regulates glucose deprivation-induced necrosis. Oncol Rep.

[17] Josefsen K, Sorensen LR, Buschard K, Birkenbach M (1999). Glucose induces early growth response gene (Egr-1) expression in pancreatic beta cells. Diabetologia.

[18] Liu C, Chen S, Wang X, Chen Y, Tang N (2014). 15d-PGJ(2) decreases PGE(2) synthesis in HBx-positive liver cells by interfering EGR1 binding to mPGES-1 promoter. Biochem Pharmacol.

[19] Symmans WF, Hatzis C, Sotiriou C, Andre F, Peintinger F, Regitnig P, Daxenbichler G, Desmedt C, Domont J, Marth C, Delaloge S, Bauernhofer T, Valero V (2010). Genomic index of sensitivity to endocrine therapy for breast cancer. J Clin Oncol.

[20] Loi S, Haibe-Kains B, Desmedt C, Lallemand F, Tutt AM, Gillet C, Ellis P, Harris A, Bergh J, Foekens JA, Klijn JG, Larsimont D, Buyse M (2007). Definition of clinically distinct molecular subtypes in estrogen receptor-positive breast carcinomas through genomic grade. J Clin Oncol.

[21] Miller WR, Larionov A (2010). Changes in expression of oestrogen regulated and proliferation genes with neoadjuvant treatment highlight heterogeneity of clinical resistance to the aromatase inhibitor, letrozole. Breast Cancer Res.

[22] Xia J, Sinelnikov IV, Han B, Wishart DS (2015). MetaboAnalyst 3.0—making metabolomics more meaningful. Nucleic Acids Res.

[23] Kamburov A, Cavill R, Ebbels TM, Herwig R, Keun HC (2011). Integrated pathway-level analysis of transcriptomics and metabolomics data with IMPaLA. Bioinformatics.

[24] Lee SH, Bahn JH, Choi CK, Whitlock NC, English AE, Safe S, Baek SJ (2008). ESE-1/EGR-1 pathway plays a role in tolfenamic acid-induced apoptosis in colorectal cancer cells. Mol Cancer Ther.

[25] Zavadova E, Vocka M, Spacek J, Konopasek B, Fucikova T, Petruzelka L (2014). Cellular and humoral immunodeficiency in breast cancer patients resistant to hormone therapy. Neoplasma.

[26] Dabydeen SA, Kang K, Diaz-Cruz ES, Alamri A, Axelrod ML, Bouker KB, Al-Kharboosh R, Clarke R, Hennighausen L, Furth PA (2015). Comparison of tamoxifen and letrozole response in mammary preneoplasia of ER and aromatase overexpressing mice defines an immune-associated gene signature linked to tamoxifen resistance. Carcinogenesis.

[27] Hilakivi-Clarke L, Warri A, Bouker KB, Zhang X, Cook KL, Jin L, Zwart A, Nguyen N, Hu R, Cruz MI, de AS, Wang X, Xuan J (2017). Effects of In Utero Exposure to Ethinyl Estradiol on Tamoxifen Resistance and Breast Cancer Recurrence in a Preclinical Model. J Natl Cancer Inst.

[28] Eid MA, Kumar MV, Iczkowski KA, Bostwick DG, Tindall DJ (1998). Expression of early growth response genes in human prostate cancer. Cancer Res.

[29] Thigpen AE, Cala KM, Guileyardo JM, Molberg KH, McConnell JD, Russell DW (1996). Increased expression of early growth response-1 messenger ribonucleic acid in prostatic adenocarcinoma. J Urol.

[30] Scharnhorst V, Menke AL, Attema J, Haneveld JK, Riteco N, van Steenbrugge GJ, van der Eb AJ, Jochemsen AG (2000). EGR-1 enhances tumor growth and modulates the effect of the Wilms’ tumor 1 gene products on tumorigenicity. Oncogene.

[31] Gaggioli C, Robert G, Bertolotto C, Bailet O, Abbe P, Spadafora A, Bahadoran P, Ortonne JP, Baron V, Ballotti R, Tartare-Deckert S (2007). Tumor-derived fibronectin is involved in melanoma cell invasion and regulated by V600E B-Raf signaling pathway. J Invest Dermatol.

[32] Tao W, Shi JF, Zhang Q, Xue B, Sun YJ, Li CJ (2013). Egr-1 enhances drug resistance of breast cancer by modulating MDR1 expression in a GGPPS-independent manner. Biomed Pharmacother.

[33] Bae SK, Bae MH, Ahn MY, Son MJ, Lee YM, Bae MK, Lee OH, Park BC, Kim KW (1999). Egr-1 mediates transcriptional activation of IGF-II gene in response to hypoxia. Cancer Res.

[34] Shan J, Balasubramanian MN, Donelan W, Fu L, Hayner J, Lopez MC, Baker HV, Kilberg MS (2014). A mitogen-activated protein kinase/extracellular signal-regulated kinase kinase (MEK)-dependent transcriptional program controls activation of the early growth response 1 (EGR1) gene during amino acid limitation. J Biol Chem.

[35] Parra E, Ortega A, Saenz L (2009). Down-regulation of Egr-1 by siRNA inhibits growth of human prostate carcinoma cell line PC-3. Oncol Rep.

[36] Ma J, Ren Z, Ma Y, Xu L, Zhao Y, Zheng C, Fang Y, Xue T, Sun B, Xiao W (2009). Targeted knockdown of EGR-1 inhibits IL-8 production and IL-8-mediated invasion of prostate cancer cells through suppressing EGR-1/NF-kappaB synergy. J Biol Chem.

[37] Yang SZ, Abdulkadir SA (2003). Early growth response gene 1 modulates androgen receptor signaling in prostate carcinoma cells. J Biol Chem.

[38] Yang SZ, Eltoum IA, Abdulkadir SA (2006). Enhanced EGR1 activity promotes the growth of prostate cancer cells in an androgen-depleted environment. J Cell Biochem.

[39] Huang RP, Fan Y, De Belle I, Niemeyer C, Gottardis MM, Mercola D, Adamson ED (1997). Decreased Egr-1 expression in human, mouse and rat mammary cells and tissues correlates with tumor formation. Int J Cancer.

[40] Shen C, Huang Y, Liu Y, Wang G, Zhao Y, Wang Z, Teng M, Wang Y, Flockhart DA, Skaar TC, Yan P, Nephew KP, Huang TH (2011). A modulated empirical Bayes model for identifying topological and temporal estrogen receptor alpha regulatory networks in breast cancer. BMC Syst Biol.

[41] Jones AC, Trujillo KA, Phillips GK, Fleet TM, Murton JK, Severns V, Shah SK, Davis MS, Smith AY, Griffith JK, Fischer EG, Bisoffi M (2012). Early growth response 1 and fatty acid synthase expression is altered in tumor adjacent prostate tissue and indicates field cancerization. Prostate.

[42] Menendez JA, Lupu R (2017). Fatty acid synthase regulates estrogen receptor-alpha signaling in breast cancer cells. Oncogenesis.

[43] Faour WH, Alaaeddine N, Mancini A, He QW, Jovanovic D, Di Battista JA (2005). Early growth response factor-1 mediates prostaglandin E2-dependent transcriptional suppression of cytokine-induced tumor necrosis factor-alpha gene expression in human macrophages and rheumatoid arthritis-affected synovial fibroblasts. J Biol Chem.

[44] Hansen PE (1994). Tolfenamic acid in acute and prophylactic treatment of migraine, a review. Pharmacol Toxicol.

[45] Kim HJ, Cho SD, Kim J, Kim SJ, Choi C, Kim JS, Nam JS, Han KK, Kang KS, Jung JY (2013). Apoptotic effect of tolfenamic acid on MDA-MB-231 breast cancer cells and xenograft tumors. J Clin Biochem Nutr.

[46] Liu X, Abdelrahim M, Abudayyeh A, Lei P, Safe S (2009). The nonsteroidal anti-inflammatory drug tolfenamic acid inhibits BT474 and SKBR3 breast cancer cell and tumor growth by repressing erbB2 expression. Mol Cancer Ther.

[47] Lee SH, Bahn JH, Whitlock NC, Baek SJ (2010). Activating transcription factor 2 (ATF2) controls tolfenamic acid-induced ATF3 expression via MAP kinase pathways. Oncogen.

[48] Kang SU, Shin YS, Hwang HS, Baek SJ, Lee SH, Kim CH (2012). Tolfenamic acid induces apoptosis and growth inhibition in head and neck cancer: involvement of NAG-1 expression. PLoS One.

[49] Brunner N, Boysen B, Jirus S, Skaar TC, Holst-Hansen C, Lippman J, Frandsen T, Spang-Thomsen M, Fuqua SA, Clarke R (1997). MCF7/LCC9: an antiestrogen-resistant MCF-7 variant in which acquired resistance to the steroidal antiestrogen ICI 182,780 confers an early cross-resistance to the nonsteroidal antiestrogen tamoxifen. Cancer Res.

[50] Schwartz-Roberts JL, Shajahan AN, Cook KL, Warri A, Abu-Asab M, Clarke R (2013). GX15–070 (obatoclax) induces apoptosis and inhibits cathepsin D- and L-mediated autophagosomal lysis in antiestrogen-resistant breast cancer cells. Mol Cancer Ther.

[51] Shajahan-Haq AN, Cook KL, Schwartz-Roberts JL, Eltayeb AE, Demas DM, Warri AM, Facey CO, Hilakivi-Clarke LA, Clarke R (2014). MYC regulates the unfolded protein response and glucose and glutamine uptake in endocrine resistant breast cancer. Mol Cancer.

[52] Sheikh KD, Khanna S, Byers SW, Fornace A, Cheema AK (2011). Small molecule metabolite extraction strategy for improving LC/MS detection of cancer cell metabolome. J Biomol Tech.

[53] Tautenhahn R, Bottcher C, Neumann S (2008). Highly sensitive feature detection for high resolution LC/MS. BMC Bioinformatics.

[54] Evans AM, DeHaven CD, Barrett T, Mitchell M, Milgram E (2009). Integrated, nontargeted ultrahigh performance liquid chromatography/electrospray ionization tandem mass spectrometry platform for the identification and relative quantification of the small-molecule complement of biological systems. Anal Chem.

[55] Gautier L, Cope L, Bolstad BM, Irizarry RA (2004). affy—analysis of Affymetrix GeneChip data at the probe level. Bioinformatics.

[56] Ritchie ME, Phipson B, Wu D, Hu Y, Law CW, Shi W, Smyth GK (2015). limma powers differential expression analyses for RNA-sequencing and microarray studies. Nucleic Acids Res.

[57] Benton HP, Want EJ, Ebbels TM (2010). Correction of mass calibration gaps in liquid chromatography-mass spectrometry metabolomics data. Bioinformatics.

[58] Smith CA, Want EJ, O’Maille G, Abagyan R, Siuzdak G (2006). XCMS: processing mass spectrometry data for metabolite profiling using nonlinear peak alignment, matching, and identification. Anal Chem.

[59] Bolstad BM, Irizarry RA, Astrand M, Speed TP (2003). A comparison of normalization methods for high density oligonucleotide array data based on variance and bias. Bioinformatics.

[60] Kuhl C, Tautenhahn R, Bottcher C, Larson TR, Neumann S (2012). CAMERA: an integrated strategy for compound spectra extraction and annotation of liquid chromatography/mass spectrometry data sets. Anal Chem.

[61] Leek JT, Storey JD (2007). Capturing heterogeneity in gene expression studies by surrogate variable analysis. PLoS Genet.

[62] Leek JT, Johnson WE, Parker HS, Jaffe AE, Storey JD (2012). The sva package for removing batch effects and other unwanted variation in high-throughput experiments. Bioinformatics.

[63] Smith CA, O’Maille G, Want EJ, Qin C, Trauger SA, Brandon TR, Custodio DE, Abagyan R, Siuzdak G (2005). METLIN: a metabolite mass spectral database. Ther Drug Monit.

[64] Wishart DS, Jewison T, Guo AC, Wilson M, Knox C, Liu Y, Djoumbou Y, Mandal R, Aziat F, Dong E, Bouatra S, Sinelnikov I, Arndt D (2013). HMDB 3.0—The Human Metabolome Database in 2013. Nucleic Acids Res.

[65] Chen EY, Tan CM, Kou Y, Duan Q, Wang Z, Meirelles GV, Clark NR, Ma’ayan A (2013). Enrichr: interactive and collaborative HTML5 gene list enrichment analysis tool. BMC Bioinformatics.

[66] Kanehisa M, Goto S, Sato Y, Kawashima M, Furumichi M, Tanabe M (2014). Data, information, knowledge and principle: back to metabolism in KEGG. Nucleic Acids Res.

[67] Milacic M, Haw R, Rothfels K, Wu G, Croft D, Hermjakob H, D’Eustachio P, Stein L (2012). Annotating cancer variants and anti-cancer therapeutics in reactome. Cancers (Basel).

[68] Kuhn M, Szklarczyk D, Pletscher-Frankild S, Blicher TH, von MC, Jensen LJ, Bork P (2014). STITCH 4: integration of protein-chemical interactions with user data. Nucleic Acids Res.

[69] Gauba R, Madhavan S, Clake R, Gusev Y (2014). Integrative analysis workflow for untargeted metabolomics in translational research. metabolomics.

[70] Cline MS, Smoot M, Cerami E, Kuchinsky A, Landys N, Workman C, Christmas R, Avila-Campilo I, Creech M, Gross B, Hanspers K, Isserlin R, Kelley R (2007). Integration of biological networks and gene expression data using Cytoscape. Nat Protoc.

[71] Romanelli S, Perego P, Pratesi G, Carenini N, Tortoreto M, Zunino F (1998). *In vitro* and *in vivo* interaction between cisplatin and topotecan in ovarian carcinoma systems. Cancer Chemother Pharmacol.

